# Meeting the mental health needs of people with the complex neurodevelopment condition, Prader-Willi syndrome: a major challenge for mental health services

**DOI:** 10.1192/j.eurpsy.2026.11686

**Published:** 2026-07-31

**Authors:** T. Holland, M. Walker

**Affiliations:** 1 Psychiatry, University of Cambridge, Cambridge; 2 Chief Executive Officer, International Prader Willi Syndrome Organisation, Windsor, United Kingdom

## Abstract

**Introduction:**

Prader-Willi Syndrome (PWS) is a rare genetically determined neurodevelopmental condition that is associated with complex physical, behavioural and neuropsychiatric phenotypes. The neuropsychiatric and behavioural phenotypes include the development of severe hyperphagia in early childhood which, if not managed, results in life-threatening obesity; a high risk of emotional outbursts and self-injurious behaviour; specific cognitive impairments; and an increased risk of mood disorders and the development of psychotic illness in the teenage years. In 2025 after 3 years of consultation IPWSO launched an interdisciplinary international Report on the mental health needs of people with PWS highlight the complexity of need, the existing interventions, the importance of quality social support, and the need for further research to develop new treatments.

**Objectives:**

1. To improve understanding of the behavioural and neuropsychiatric phenotype of PWS and of the mental health needs of children and adults with thge syndrome, and the needs of their families.
2. To inform about interventions to meet the mental health needs of people with the syndrome and that of their families.
3. To encourage debate about the role of advocacy organisations, such as IPWSO, and the design of services.

**Methods:**

The conclusions in the Report were based on published research and systematic reviews, experiences described by people with PWS and their families, expert opinion, and through virtual and face-to-face consultations, and workshops. This presentation will draw upon this global Report.

**Results:**

An increasing body of research has idenfied and characterised the bevioural and mental health needs of people with PWS. Understanding has improved but specific effective treatments are lacking although trials for treatments of hyperphagia are underway and one treatment has been approved. Behaviour and mental health needs are a major concern for families yet services in all countries are severly lacking.

**Conclusions:**

The behavioural and mental health needs of people with PWS result from the interactions between biological vulnerabilities associated with having PWS and living in a changing and demanding environment. Interventions can be at different levels including directly working with peoiple with PWS, supporting families and paid professionals to manage the food environment and to more effectively manage other behaviours, and specific psychological and psychiatric treatments targeting specific aspects of the PWS behavioural and neuropsychiatric phenotype.

**Disclosure of Interest:**

T. Holland Consultant of: Advisor to pharmaceutical companies developing new treatments - Euros 5,000 to 7000 a year, M. Walker: None Declared

